# Impact of acute kidney injury and dysnatremia on length of stay in infants after cardiac surgery

**DOI:** 10.1007/s00467-025-06846-7

**Published:** 2025-06-14

**Authors:** Jonas Rønne Kronborg, Rasmus Bo Lindhardt, Niels Vejlstrup, Line Marie Holst, Klaus Juul, Morten Holdgaard Smerup, Jakob Gjedsted, Hanne Berg Ravn

**Affiliations:** 1 Department of Cardiothoracic Anesthesiology, Rigshospitalet, Copenhagen University Hospital, Copenhagen, Denmark; 2 Faculty of Health and Medical Sciences, University of Copenhagen, Copenhagen, Denmark; 3 Department of Anesthesiology and Intensive Care, Odense University Hospital, Odense, Denmark; 4 Department of Clinical Research, University of Southern Denmark, Odense, Denmark; 5 Department of Cardiology, Rigshospitalet, Copenhagen University Hospital, Copenhagen, Denmark; 6 Department of Pediatric Cardiology, Rigshospitalet, Copenhagen University Hospital, Copenhagen, Denmark; 7 Department of Cardiothoracic Surgery, Rigshospitalet, Copenhagen University Hospital, Copenhagen, Denmark; 8 Department of Clinical Medicine, University of Copenhagen, Copenhagen, Denmark

**Keywords:** Acute kidney injury, Cardiac surgery, Critical care, Congenital heart disease, Hyponatremia, Hypernatremia

## Abstract

**Background:**

Acute kidney injury (AKI) and dysnatremia following pediatric cardiac surgery are common conditions associated with worse outcomes. While the multifactorial etiology of AKI is well-known, the role of concomitant dysnatremia is limited. This study aims to describe the occurrence of AKI, its association with the length of stay in the intensive care unit (ICU-LOS), and the impact of dysnatremia in the context of AKI.

**Methods:**

Retrospective study comprising 228 congenital heart procedures in 213 infants at Rigshospitalet, Copenhagen, Denmark, from 2017 to 2019. AKI development was evaluated separately in neonates and infants > 1 month and its impact on ICU-LOS. Risk factors for AKI were analyzed across age groups using the univariate and multivariate logistic regression analysis.

**Results:**

AKI occurred in 61% of neonates and 62% of infants. Severity was comparable across age groups, except for KDIGO-stage 3, where seven out of eight children treated with peritoneal dialysis were neonates. Urine output was well-preserved despite AKI development, but children with AKI required more than double the furosemide dose. In multivariate analysis, prolonged cardiopulmonary bypass (CPB) duration, higher furosemide doses, and hypernatremia were independently associated with AKI. AKI was only associated with prolonged ICU-LOS in infants, while hyponatremia was associated with prolonged ICU-LOS in all individuals with AKI.

**Conclusions:**

AKI occurs frequently in neonates and infants after congenital heart surgery but is only associated with prolonged ICU-LOS in infants. The co-occurrence of AKI and hyponatremia leads to longer ICU-LOS in both neonates and infants. Independent predictors of AKI were prolonged CPB duration, hypernatremia, and reduced furosemide sensitivity.

**Graphical Abstract:**

**Figure:**
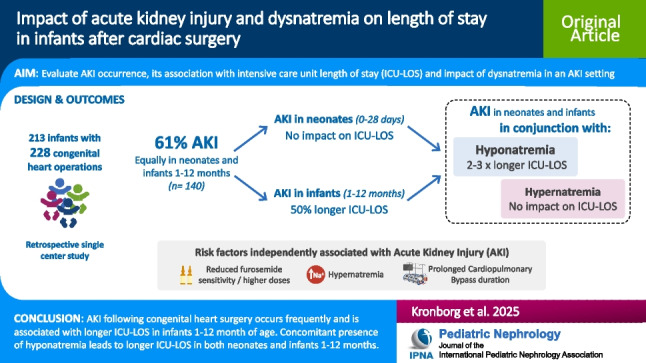
A higher resolution version of the Graphical abstract is available as Supplementary information

**Supplementary Information:**

The online version contains supplementary material available at 10.1007/s00467-025-06846-7.

## Introduction

Acute kidney injury (AKI) is common after pediatric congenital cardiac surgery and has previously been associated with increased morbidity and mortality [1]. Development of AKI has been observed in the range of 20–70% after pediatric cardiac surgery, depending on patient age, procedural complexity, cardiopulmonary bypass (CPB) duration, perioperative management, as well as criteria for determination of AKI [1–8]. AKI criteria have included various definitions over the recent decades, including AKIN, pRIFLE criteria, and more recently, the KDIGO criteria, where the individual criteria have advantages and disadvantages as well as variable associations with outcome [1, 9]. Given the fact that AKI is often associated with fluid overload, this may contribute to challenges in diagnostic precision but also potentially lead to electrolyte disturbances. We have previously shown that dysnatremia is one of the most common electrolyte disturbances following congenital heart surgery, and dysnatremia is associated with increased intensive care unit length of stay (ICU-LOS) [10]. However, little is known about the relationship between dysnatremia and the development of AKI.

Aim of the present study was to describe the occurrence of AKI in neonates and infants older than one month after cardiac surgery. Furthermore, we sought to confirm or defer the association between AKI and length of stay in the intensive care unit (ICU) previously reported, while also evaluating the contribution of dysnatremia in the setting of AKI.

## Methods

This is an observational single-center study with retrospective data collection conducted at Rigshospitalet, Copenhagen University Hospital, Denmark, between January 1, 2017 and July 31, 2019. Pediatric heart surgery in Denmark was in 2016 consolidated into a single national center at Rigshospitalet. Following this centralization, a retrospective evaluation of institutional perioperative practices, including fluid therapy, electrolyte disturbances, and their impact on short-term outcomes after congenital heart surgery, was initiated. The study was conducted in accordance with the Declaration of Helsinki after approval from the Institutional Review Board (IRB) at Rigshospitalet, Copenhagen University Hospital, Denmark (21026067). Informed consent was waived by the IRB due to the retrospective study design. Detailed descriptions of data sources, perioperative, and anesthetic management within this cohort can be found in previous publications [10, 11].

### Study population

All infants undergoing surgery for congenital heart disease (CHD) in Denmark during this period were assessed for eligibility for this study. To qualify for inclusion in this study, infants had to meet the criteria: surgery for CHD with the use of CPB. Infants were excluded from the analysis if their surgery was not RACHS-1 applicable, if they received extracorporeal membrane oxygenation, died before ICU discharge, underwent surgery for a non-cardiac malformation along with the CHD surgery, had less than 24 h of postoperative follow-up, developed complicated dysnatremia (both hyper- and hyponatremia either intraoperatively or within the first 48 postoperative hours), or there was an absence of blood samples.

In infants undergoing multiple surgical procedures within 12 months of age, an additional surgical procedure was only included if the procedure was not performed during the same admission. In other words, surgical procedures were considered independent events if the infant was discharged and more than 30 days had elapsed before the second operation. For improved readability, we have defined the two age groups at the time of surgery as (1) neonates as individuals 28 days or younger and (2) infants as children below the age of 1 year but older than 28 days.

### Timeline

Data were collected during the preoperative, intraoperative, and postoperative periods. The intraoperative period was defined as the time from arrival in the operating room until ICU admission. Postoperative day 0 (POD0) was from ICU admission until 6:00 am the following morning, and postoperative day 1 (POD1) was from 6:00 am until 6:00 am the following day. The first 48 postoperative hours were defined as the time from ICU admission until the end of POD1. ICU-LOS was calculated from the moment of ICU admission until discharge to a pediatric ward.

### Outcome data

The primary outcome was ICU-LOS in relation to AKI and dysnatremia development during the first 48 postoperative hours. A secondary outcome was to describe the occurrence and risk factors associated with the development of AKI.

AKI was categorized according to the KDIGO definition including the neonatal modifications if applicable (Supplementary Table S1) [12–17]. AKI was defined as a serum creatinine (s-creatinine) increase of ≥ 0.3 mg/dl (≥ 26.5 µmol/l), a ≥ 50% increase in s-creatinine from baseline, or an average urinary output (UOP) of less than 1 ml/kg/h per hour, or the use of peritoneal dialysis. Individuals receiving peritoneal dialysis were categorized as KDIGO stage 3. AKI was calculated for each 24-h period through to postoperative day 7, and patients were categorized based on their highest AKI stage. The UOP criterion was only used until the end of POD1 and was based on daily totals in both infants and neonates. In infants, the baseline s-creatinine was the lowest obtained value 14 days prior to surgery. In neonates, the baseline was the lowest s-creatinine measurement preceding the analyzed sample (rolling baseline), as s-creatinine naturally decreases during the first weeks after birth due to the physiological kidney maturation in neonates [18].

Hypernatremia and hyponatremia data were recorded during the first 48 postoperative hours after surgery and were defined as plasma sodium levels exceeding 145 mmol/L and below 135 mmol/L, respectively, in one or more blood samples. All blood samples were examined for potential dilution and excluded from analysis if (1) unphysiological electrolyte concentrations were present (potassium or chloride exceeding 9 and 140 mmol/L, respectively, or sodium below 110 mmol/L) or (2) unphysiological fluctuations in plasma sodium, such as a sudden increase or decrease exceeding 10 mmol/L, only to return to previous values within 30 min. All sodium measurements from each individual infant were analyzed in this manner by visually plotting them against their corresponding time points.

Daily fluid balance was calculated using the input/output methodology, with intraoperative fluid included in the fluid balance on POD0 [19]. Cumulative daily fluid balance (cumulative-FB) was calculated as a percentage change from the preoperative weight, assuming a gain of 10 ml/kg equals a 1% increase in weight. The theoretical intravenous free water administration was calculated as previously described [10, 11].

### Data source

In brief, data were extracted from the electronic patient record and entered in REDCap (Research Electronic Data Capture). To ensure data accuracy and minimize potential data entry errors, all manually inputted data were double entered into REDCap by J.R.K and/or R.B.L., with a minimum of 1 month separating the entries. Blood samples were analyzed at the Department of Clinical Biochemistry at Rigshospitalet or locally using a blood gas analyzer (ABL800 Flex; Radiometer, Copenhagen, Denmark). All blood samples were collected at the discretion of the treating clinicians, and all available blood samples prior to surgery and during the first seven postoperative days were included in this study. Clinical variables were recorded only intraoperatively and postoperatively on POD0 and POD1.

### Statistical analysis

Continuous variables are reported as either mean ± standard deviation (SD) or median and interquartile range (IQR). Categorical variables are reported as numbers and frequencies (%). The distribution of data was evaluated visually with histograms. Two group comparisons were performed using either the *t*-test or the Wilcoxon rank-sum test, depending on the distribution of the data. Categorical variables were compared using chi-squared or *Fisher’s exact *test. The Kruskal–Wallis *H*-test was applied to overall comparisons between multiple groups and to describe overall trends in data. If significant (*p* < 0.05), the Mann–Whitney *U*-test was applied in intergroup comparisons. Univariate and multivariate logistic regression analyses were performed on the total study population to increase the statistical power and increase the number of variables to match the number of events (at least ten events per variable). Only variables that were significantly associated with postoperative AKI (*p* < 0.05) were included in a multivariate logistic regression model.

All tests were two-sided, and *p* < 0.05 was considered significant. All statistical analyses were performed using R (version 1.1.4) and R Studio (version 4.3.2).

## Results

A total of 289 infants who underwent 323 operations were assessed for eligibility. Of these, 95 operations performed on 76 infants were excluded (Supplementary Figure S1), leaving 228 surgical procedures in 213 individuals. Among the included children, 134 (58.8%) were male and 61 (26.8%) were neonates. At the time of surgery, the median age and weight were 8 days and 3.5 kg in neonates and 127 days and 5.6 kg in infants, respectively (Table 1). Eight infants (3.5%), of which seven were neonates, required postoperative peritoneal dialysis. Patient characteristics and periprocedural data are presented in Table 1. CPB duration was significantly longer in infants with AKI, but not in neonates (Table 1).

**Table 1:** Demographics and patient characteristics

		Neonate		Infant		Overall
		No AKI *n* = *23*	AKI *n* = *38*	No AKI *n* = *65*	AKI *n* = *102*	*n* = 228
Age (days)		7.0 (6.0–10.0)	8.0 (6.0–10.0)	108.0 (78.0–168.0)	142.0 (84.2–203.5)	91.5 (19.2–174.8)
Weight (kg)		3.6 (3.4–3.9)	3.5 (3.1–3.9)	5.3 (4.4–6.3)	6.0^†^ (4.7–7.4)	4.8 (3.8–6.4)
Sex (male)		13 (56.5%)	27 (71.1%)	36 (55.4%)	58 (56.9%)	134 (58.8)
CPB time (minutes)		120.0 (104.0–162.0)	142.0 (113.0–190.0)	78.0 (60.0–99.0)	117.5^‡^ (83.5–157.5)	105 (75.8–153.0)
Cross clamp time (minutes)		69.5 (56.0–79.0)	71.0 (48.2–115.0)	50.0 (35.0–61.5)	71.0^‡^ (49.0–102.8)	62.0 (44.0–90.0)
RACHS-1 category	1–2	0 (0%)	2 (5.3%)	50 (76.9%)	76 (74.5%)	128 (56.1%)
	3–4	22 (95.7%)	33 (86.8%)	15 (23.1%)	26 (25.5%)	96 (42.1%)
	5–6	1 (4.3%)	3 (7.9%)	0 (0%)	0 (0%)	4 (1.8%)

*AKI* acute kidney injury, *CPB* cardiopulmonary bypass, RACHS-1 Risk Adjustment for Congenital Heart Surgery 1

Data presented as median (IQR) or *n* (%). Overall comparison listed in the AKI column (separately for neonates and infants): *p*-value: ^†^ ≤ 0.01, ^‡^ ≤ 0.001

**Fig. 1:**
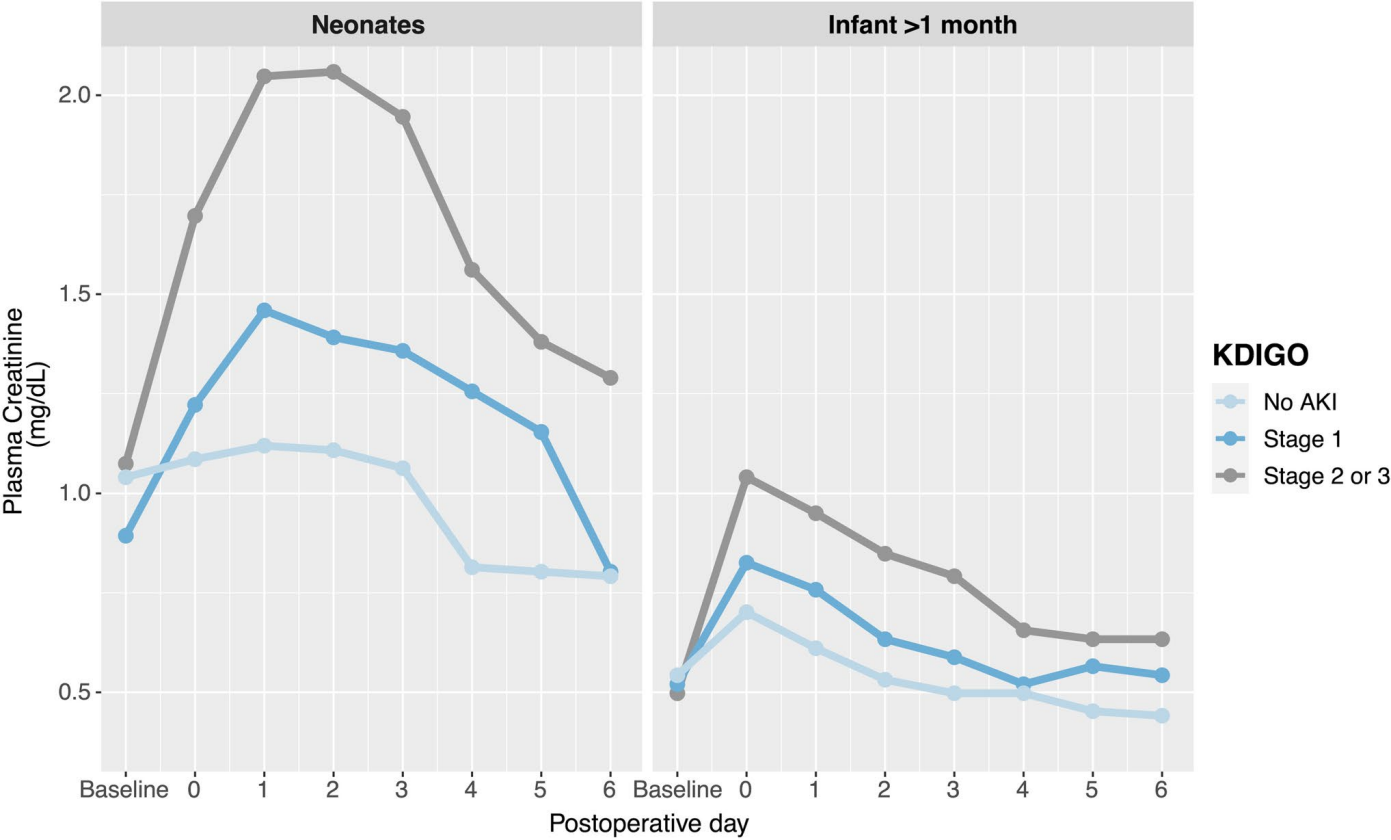
Progression of median plasma creatinine postoperatively according to the severity of acute kidney injury (AKI). Baseline values were included. Samples were predominantly collected at the end of each postoperative day. Moderate and severe AKI (KDIGO stages 2 and 3) were combined due to a low number of stage 3 infants. AKI, acute kidney injury; KDIGO, kidney disease improving global outcomes

Postoperative AKI occurrence was similar in neonates and infants, with 62% and 61%, respectively. The development of AKI peaked during POD0 in infants and POD1 in neonates (Fig. 1). There was no association between AKI and RACHS-1 classification, neither in neonates nor in infants.

In neonates, the development of AKI was significantly associated with the volume of isotonic glucose and blood products, in particular red blood cells as well as furosemide dose (*p* < 0.01; Table 2). In infants, AKI was associated with intraoperative bleeding, cryoprecipitate volume, and furosemide dose (*p* < 0.05; Table 3). Diuresis was preserved in all children with an average diuresis above 6 ml/kg/hour in neonates and 4 ml/kg/hour in infants, irrespective of AKI occurrence (Tables 2 and 3). Despite a higher dose of diuretics in children with AKI in both age groups, there was no significant difference in fluid balance on POD0 and 1 in children with and without AKI (Tables 2 and 3).

**Table 2:** In neonates, accumulated volume/doses intra- and postoperatively (first up to 48 postoperative hours)

	No AKI *n* = *23*	AKI *n* = *38*	*p*-value
Darrow glucose (ml kg^−1^)	70.0 (58.4–84.2)	61.7 (44.9–81.8)	0.137
IV glucose (ml kg^−1^)	29.9 (9.6–40.5)	56.7 (22.8–75.7)	**0.009**
Free water intake (ml kg^−1^)	82.0 (57.2–103.6)	103.1 (73.3–133.2)	0.127
Blood products (ml kg^−1^)	49.4 (34.5–90.9)	80.7 (61.2–133.4)	**0.019**
RBC transfusions (ml kg^−1^)	24.7 (11.4–44.1)	41.3 (31.7–71.1)	**0.008**
Cryoprecipitate (ml kg^−1^)	14.3 (8.0–19.0)	18.6 (14.3–30.5)	**0.049**
Pooled platelets (ml kg^−1^)	13.7 (10.1–24.7)	16.9 (12.8–36.2)	0.287
Intraoperative bleeding (ml kg^−1^ h^−1^)	7.9 (3.8–17.0)	15.2 (7.2–24.8)	0.093
Postoperative bleeding (ml kg^−1^ h^−1^)	1.0 (0.8–1.3)	1.4 (1.1–2.1)	**0.015**
Furosemide dose (mg kg^−1^)	6.9 (2.4–16.7)	17.4 (7.2–21.9)	**0.015**
Diuresis^§^ (ml kg^−1^ h^−1^)	6.5 (5.9–7.8)	7.2 (5.6–9.1)	0.602
Cumulative-FB POD0 (%)	0.1% (− 3.3–4.6)	0.6% (− 2.9–5.8)	0.590
Cumulative-FB POD1 (%)	−3.1% (− 6.9– − 0.2)	− 3.0% (− 8.4–1.9)	0.959

*AKI* acute kidney injury, *Cumulative-FB* cumulative fluid balance, *IV* intravenous, *POD* postoperative day, *RACHS-1* Risk Adjustment for Congenital Heart Surgery 1, *RBC* red blood cells

All data presented as median (IQR)

^**§**^Only registered postoperatively. Blood products comprise the total volume of red blood cells, cryoprecipitate, and pooled platelets

**Table 3:** In infants, accumulated volume/doses intra- and postoperatively (first up to 48 postoperative hours)

	No AKI *n* = *65*	AKI *n* = *102*	p-value
Darrow glucose (ml kg^−1^)	52.7 (36.8–77.3)	57.3 (41.8–76.0)	0.646
IV glucose (ml kg^−1^)	10.2 (4.1–24.0)	13.5 (5.1–29.2)	0.336
Free water intake (ml kg^−1^)	53.2 (37.1–76.4)	60.0 (39.8–81.6)	0.344
Blood products (ml kg^−1^)	30.6 (19.1–46.9)	32.1 (20.5–53.5)	0.429
RBC transfusions (ml kg^−1^)	15.0 (9.4–27.5)	19.2 (7.9–28.8)	0.618
Cryoprecipitate (ml kg^−1^)	5.6 (0.0–9.4)	8.3 (0.0–16.0)	**0.018**
Pooled platelets (ml kg^−1^)	9.0 (4.2–15.3)	8.9 (2.5–17.3)	0.853
Intraoperative bleeding (ml kg^−1^ h^−1^)	3.4 (2.3–6.7)	5.6 (3.0–11.4)	**0.007**
Postoperative bleeding (ml kg^−1^ h^−1^)	0.9 (0.7–1.3)	0.9 (0.7–1.4)	0.872
Furosemide dose (mg kg^−1^)	2.6 (0.8–7.3)	7.9 (3.3–16.9)	** < 0.001**
Diuresis^§^ (ml kg^−1^ h^−1^)	4.0 (3.4–5.1)	4.5 (3.6–5.5)	0.171
Cumulative-FB POD0 (%)	1.2% (− 1.4–3.4)	1.7% (0.2–4.2)	0.054
Cumulative-FB POD1 (%)	− 0.2% (− 2.2–2.0)	0.5% (− 1.9–3.8)	0.321

*AKI* acute kidney injury, *Cumulative-FB* cumulative fluid balance, *IV* intravenous, *POD* postoperative day, *RACHS-1* Risk Adjustment for Congenital Heart Surgery 1, *RBC* red blood cells

All data presented as median (IQR)

^**§**^Only registered postoperatively. Blood products comprise the total volume of red blood cells, cryoprecipitate, and pooled platelets

A univariate analysis of risk factors in all cases demonstrated a significant association with the same risk factors, hypo- and hypernatremia; but, after a multivariate correction, only CPB duration, hypernatremia, and furosemide dose were significantly associated with AKI development (Table 4).

**Table 4:** Uni- and multivariate analysis on postoperative AKI in neonates and infants (*n* = 188)

	Univariate analysis		Multivariate analysis	
Variable	Odds ratio (95% CI)	*p*-value	Odds ratio (95% CI)	*p*-value
Age at surgery (weeks)	1.01 (0.99–1.04)	0.196		
Prematurity (yes)	0.98 (0.41–2.44)	0.956		
Neonate (yes)	0.95 (0.51–1.73)	0.867		
Weight (kg)	1.17 (1.00–1.37)	0.056		
Sex (male)	0.81 (0.47–1.40)	0.453		
RACHS1 (pr category)	1.18 (0.22–5.59)	0.833		
CPB duration (pr 15 min)	1.22 (1.12–1.35)	** < 0.001**	1.18 (1.05–1.35)	**0.009**
Dysnatremia				
Normonatremia	Ref		Ref	
Hyponatremia	2.23 (1.17–4.34)	**0.016**	2.27 (0.95–5.56)	0.076
Hypernatremia	2.56 (1.33–5.05)	**0.006**	2.57 (1.09–6.24)	**0.020**
RBC transfusion (ml kg^−1^)	1.01 (1.00–1.03)	**0.029**	1.01 (0.99–1.03)	0.908
Cryoprecipitate (ml kg^−1^)	1.03 (1.01–1.06)	**0.021**	0.98 (0.94–1.03)	0.173
Polled platelets (ml kg^−1^)	1.01 (0.99–1.03)	0.354		
Intraoperative bleeding (ml kg^−1^ h^−1^)	1.05 (1.02–1.09)	**0.009**	1.03 (0.97–1.09)	0.157
Postoperative bleeding (ml kg^−1^ h^−1^)	1.35 (0.95–2.00)	0.111		
Furosemide (mg kg^−1^)	1.09 (1.05–1.14)	** < 0.001**	1.07 (1.02–1.14)	**0.027**
Postoperative diuresis (ml kg^−1^ h^−1^)	1.06 (0.93–1.21)	0.371		
Cumulated fluid balance end of POD0, (per %)	1.08 (1.02–1.14)	**0.013**	1.07 (0.99–1.17)	0.067
Cumulated fluid balance end of POD1, (per %)	1.02 (0.97–1.07)	0.414		
Human albumin (g kg^−1^)	1.08 (0.98–1.20)	0.138		
HCO (mg kg^−1^)	1.02 (0.96–1.09)	0.611		
IV glucose (ml kg^−1^ h^−1^)	1.01 (1.01–1.02)	**0.017**	1.00 (0.99–1.02)	0.59
Free water load (ml kg^−1^ h^−1^)	1.18 (0.87–1.64)	0.293		

*CI* confidence interval, *CPB* cardiopulmonary bypass, *RBC* red blood cells, *Ref* reference, *POD* postoperative day, *HCO* sodium bicarbonate, *IV* intravenous

Forty cases were excluded due to variables missing at random (see Supplementary Table S5)

**Fig. 2:**
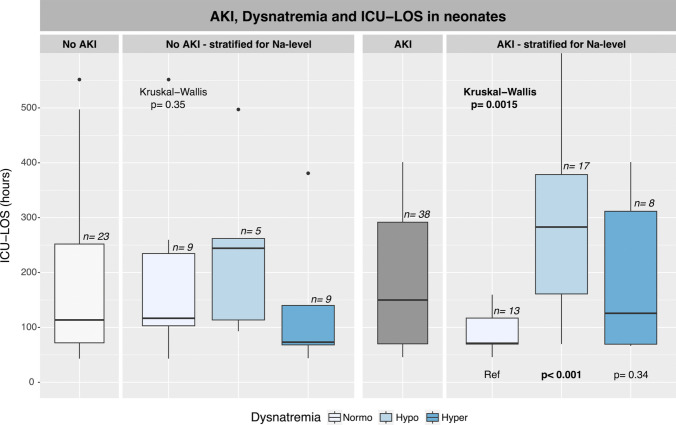
In neonates, ICU-LOS stratified according to AKI and dysnatremia in infants in the first 48 postoperative hours. Normonatremia was used as reference for intergroup comparisons if overall testing was significant. Boxplots display median values, IQR, and outliers. AKI, acute kidney injury; ICU-LOS, intensive care unit length of stay; Na, plasma sodium; Ref, reference

**Fig. 3:**
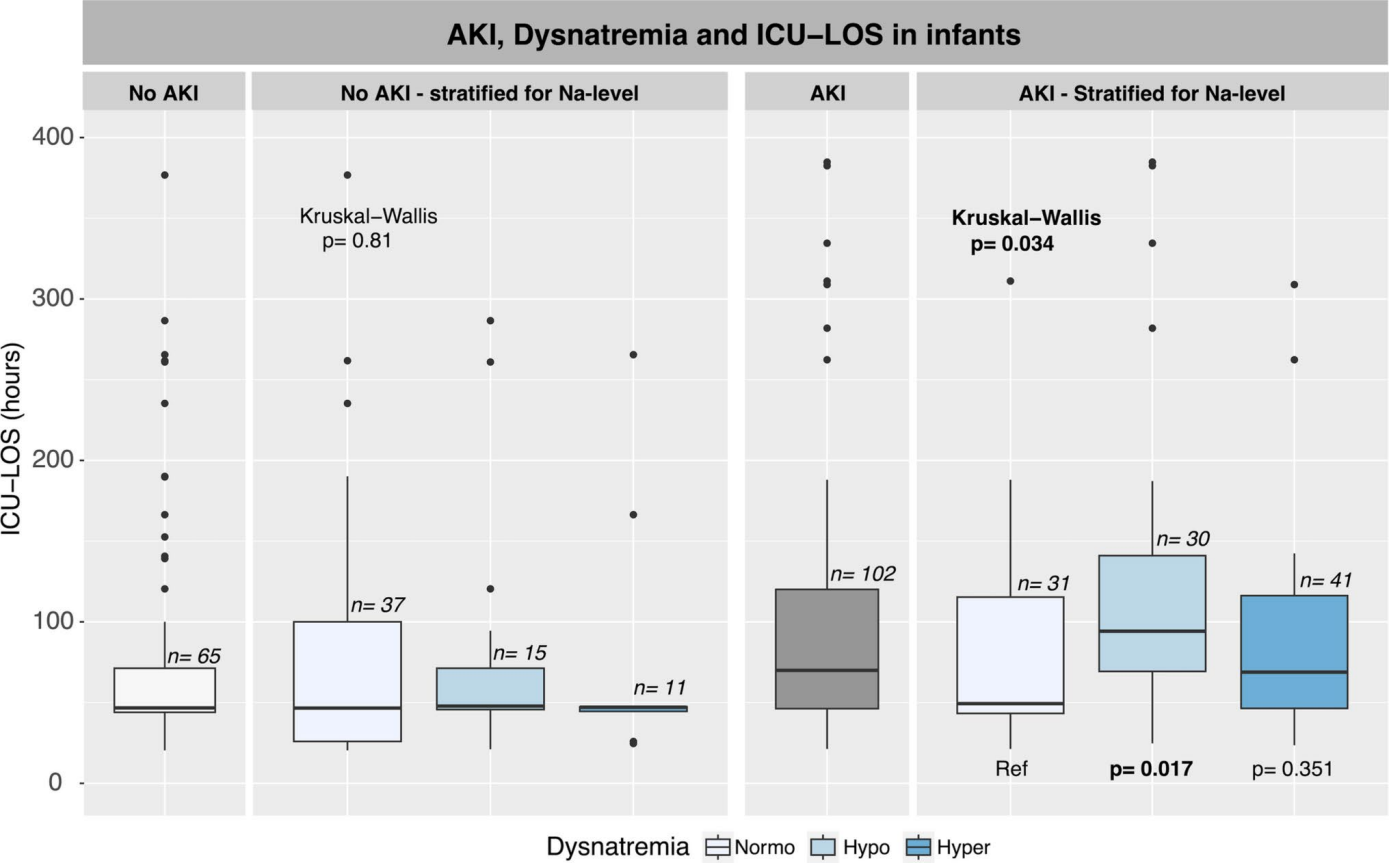
In infants, ICU-LOS stratified according to AKI and dysnatremia in infants in the first up to 48 postoperative hours. Normonatremia was used as reference to intergroup comparisons if overall testing was significant. Boxplots display median values, IQR, and outliers. AKI, acute kidney injury; ICU-LOS, intensive care unit length of stay; Na, plasma sodium; Ref, reference

In neonates (Fig. 2), AKI was not associated with increased ICU-LOS (114 [IQR 72–252 h] vs. 150 [IQR 70–292 h]; n.s.). In infants (Fig. 3), however, AKI was associated with a 50% longer ICU-LOS (47 [IQR 44–71 h] vs. 70 ([IQR 46–120 h]; *p* ≤ 0.01). The combination of AKI and hyponatremia led to a significant increase in LOS in both neonates and infants (Figs. 2 and 3).

## Discussion

Postoperative AKI was observed in more than 60% of all cases and with comparable frequency in neonates and infants. AKI development was associated with longer ICU-LOS in infants, but not in neonates. AKI, with concomitant hyponatremia, was associated with increased ICU-LOS in both age groups, but not hypernatremia.

Kidney function and s-creatinine levels change rapidly during the first weeks of life, and the kidneys continue to mature until 12–18 months of age [16, 20]. Creatinine levels normally decline steadily after birth. This leads to the hypothetical issue that a steady creatinine may indicate AKI in newborns, since the natural development within the first weeks of life is a decline [21]. Creatinine levels immediately after birth are influenced by maternal kidney function, birth weight, and gestational age at the time of birth [20, 22]. In the present study, 50% of neonates were less than 1 week old at the time of surgery, which is reflected in a nearly twice as high preoperative s-creatinine value in the neonatal group (0.95 vs. 0.52 mg/dL). Due to a higher baseline creatinine, an increase of 50% is much less likely to occur with a reference value of 1.0 mg/dL compared to a baseline level of 0.5 mg/dL. These circumstances could lead to an underestimation/misclassification of AKI in neonates, especially during the first weeks of life [17]. The current KDIGO definition remains the gold standard and has been shown to provide a more conservative estimate of AKI and a stronger association with outcome [1]. We chose to analyze risk factors for AKI development in two separate age groups, since patient population, surgical complexity, and perioperative management are quite different in neonates and infants above 1 month. RACHS-1 category was mainly 3–4 in neonates and 1–2 in infants, which is also reflected in the CPB duration. Similarly, perioperative management differed significantly between age groups with more than twice the volume of blood products, isotonic glucose, human albumin, and furosemide in neonates (Supplementary Table S2). However, to increase statistical power and the number of relevant variables, we used the entire study population in the multivariate analysis.

Challenges in defining neonatal AKI are also reflected in the present study [16, 17]. The impact of AKI on length of stay (LOS) cannot be compared head-to-head across age groups, since the median LOS in infants with and without AKI was 47 and 70 h, respectively, whereas the median LOS in neonates with and without AKI was 114 and 150 h, respectively, indicating that risk factors for prolonged LOS comprise other issues aside from AKI in neonates. This may also explain the variable association of AKI with LOS in previous studies with mixed age groups [5, 6]. Of note, only advanced KDIGO AKI has been associated with prolonged LOS in selective neonatal studies [3, 4, 8] but not consistently [23]. While the KDIGO classification remains the gold standard for defining AKI, emerging data suggest that AKI should be understood as a heterogeneous syndrome with varying pathophysiology and outcomes [24]. This is particularly relevant in the setting of pediatric cardiac surgery, where transient elevation in perioperative serum creatinine may occur without this reflecting true tubular injury [24]. Ongoing research is therefore currently exploring whether sub-phenotypes of AKI, potentially identified through novel biomarkers such as NGAL, may offer more reliable detection of AKI that overcomes the limitations of serum creatinine and correlates more closely with clinical outcomes [16, 24, 25].

The pathophysiology of AKI in cardiac surgery is complex and multifactorial. Perioperative characteristics, like low cardiac output syndrome, are important risk factors, and the vasoactive inotropic score has been associated with the development of postoperative AKI. Unfortunately, we could not obtain vasoactive inotropic scores due to technical issues, and we used the isotonic glucose volume as a surrogate marker since it is exclusively applied as a carry-on solution. The more than twofold increase in glucose volume could indicate more cardiac instability in neonates with AKI. In agreement with previous reports, the RACHS-1 category had no association with AKI in the present study [25], whereas others have reported significant associations [4, 5], but this may relate to differences between the study populations. Hemodynamic alterations in the perioperative period, especially bleeding, can lead to hypovolemia and the risk of renal hypoperfusion. Bleeding or transfusion of red blood cells has been associated with an increased risk of AKI [5, 26], which agrees with observations in the present study. Even though not fully understood, inflammation has a decisive effect on the development of AKI after cardiac surgery. The use of CPB stimulates an increase in both pro- and anti-inflammatory cytokines and chemokines, where the increase in mediators is partly related to the duration of CPB [27, 28], thereby contributing to the association between CPB duration and AKI development as seen in the present study, and was also previously reported [4–6, 8, 9]. The inflammation also leads to capillary leakage and an increase in the interstitial pressure, which in turn results in reduced renal perfusion with the subsequent risk of ischemia of the renal medulla [28]. Capillary leakage treated with additional fluid administration to maintain intravascular volume can lead to fluid overload if reduced urine output occurs concomitantly. Inflammation also causes leucocyte and platelet accumulation leading to kidney tubular damage. Furthermore, the contact between red blood cells and the circuit and oxygenator surfaces may lead to hemolysis with an increase in reactive oxygen species causing renal vasoconstriction; precipitation and accumulation of free hemoglobin in the kidney can also lead to tubular injury [28]. The association between transfusion of red blood cells and AKI may partly be related to reduced oxygen carrying capacity in bank blood and more fragile erythrocytes with hemolysis [27].

The interplay between fluid overload and AKI in the context of CPB is complex. These factors often occur simultaneously and may initiate a self-perpetuating cycle of worsening fluid accumulation, tissue edema, impaired circulation, and progressive kidney dysfunction [29, 30]. Their coexistence often makes it difficult to disentangle the individual contributors in fluid imbalance. Nevertheless, fluid overload has almost consistently been associated with AKI development after cardiac surgery [3, 6, 23, 31]. Furosemide responsiveness has been used to predict the development of AKI and, in agreement with the present study, reduced sensitivity to diuretic stimulation with loop diuretics was an indicator of AKI [32, 33]. In the present study, aggressive diuretic stimulation resulted in more than twice the average dose of furosemide to obtain a comparable 24-h diuresis and equal fluid balances when comparing children with and without AKI.

Dysnatremias (both hypernatremia and hyponatremia) are common following congenital heart surgery [10], and deviations outside normal sodium levels have been associated with increased morbidity and ICU-LOS [10, 31, 34]. The relationship between dysnatremia and AKI has not been clarified. Ontenado et al. demonstrated, in agreement with the present study, that hypernatremia within the first 48 h was the most common dysnatremia in children with AKI, occurring in 39% [31], comparable to the present study (40%). In contrast, only 21% of neonates with AKI developed hypernatremia within 48 h, indicating variable etiologies to AKI in the two age groups. Hyponatremia was the most common sodium disturbance in neonates with AKI (45%), which can reflect a higher vasoactive inotropic score but also more severe fluid overload. However, no statistical difference was observed between fluid balances in children with and without AKI, though substantial interindividual variations were observed.

Interestingly, hyponatremia was associated with prolonged LOS in ICU in both neonates and infants with AKI. Interpretation of sodium deviations is hampered by the fact that the sodium level will be influenced both by an increased sodium load in medication (i.e., bicarbonate) and transfusion of blood products, but also infusion of hypotonic crystalloids like isotonic glucose [11] or by reduced diuresis/dilution due to fluid overload and capillary leakage. Unfortunately, we do not have any data on excretion of sodium in the urine, which could contribute to an understanding of the sodium deviations. Despite the fact that urine volume was comparable in children with and without AKI, we cannot determine if urine sodium concentration is the same in children with and without AKI. Furosemide inhibits the sodium potassium chloride cotransporter in the thick ascending limb of the loop of Henle and increases water and sodium excretion; but in case of loop diuretic resistance, a higher dose may be necessary to obtain the same sodium excretion, and thus also increase diuresis [35]. This could explain the observed higher furosemide dose in children with AKI but also the association with hyponatremia and LOS in the ICU since fluid overload otherwise would have led to prolonged LOS. Nonetheless, the present study emphasizes the unique combination of overlapping risk factors for the development of dysnatremia and AKI and their mutual relationship.

## Limitations

Due to the retrospective, single-center study design, it has limited generalizability, and we can only demonstrate associations rather than causality. The study is also limited by small numbers in subgroups, especially in relation to dysnatremia across age and AKI strata. This substantially increases the risk of committing type II errors. In particular, the limited number of cases in the neonatal group may have reduced the statistical power to demonstrate an association with ICU-LOS. Future studies with larger sample sizes should reexplore this association. Children who died before discharge were excluded to avoid survival bias. This may have excluded important information on dysnatremia and AKI. AKI was classified according to the KDIGO classification without fluid balance correction. We did calculate AKI occurrence after fluid balance adjustment, but the corrected creatinine levels did not change AKI occurrence or severity. Due to discharge from the ICU, fluid balance and urine output data were not available beyond POD1 in 36% of individuals, which potentially could have changed the frequency of AKI. Including a vasopressor-inotropic score would have strengthened the analysis. Isotonic glucose was exclusively used as a carrier solution for inotropic agents, but since the volume infused does not reflect the potency of inotrope or vasopressor, it is uncertain how well it reflects the severity of cardiac instability.

In conclusion, AKI after congenital heart surgery is common and was observed equally frequently in neonates and infants older than 1 month. AKI development was associated with prolonged LOS in the ICU in infants, but not in neonates. Dysnatremia was common in the presence of AKI, but only hyponatremia and AKI led to a significant prolongation of LOS in the ICU. Prolonged CPB duration, hypernatremia, and reduced furosemide sensitivity were independent predictors of AKI.

## Supplementary Information

Below is the link to the electronic supplementary material.

Supplementary file (DOCX 145 KB)

Graphical abstract (PPTX 432 KB)

## Data Availability

The data that support the findings of this study are available from the corresponding author upon reasonable request.
