# Supplementary material for: Impact of acute kidney injury and dysnatremia on length of stay in infants after cardiac surgery
Source: Pediatr Nephrol. 2025 Jun 14;40(10):3281–91. doi: 10.1007/s00467-025-06846-7 (PMC12402015; doi:10.1007/s00467-025-06846-7)
Supplement: Supplementary file 1 — Supplementary file (DOCX 145 KB) [file 467_2025_6846_MOESM1_ESM.docx]

**Supplementary Material**

**Impact of acute kidney injury and dysnatremia on length of stay**

**in infants after cardiac surgery**

Kronborg JR^1,2^, Lindhardt RB^3,4^, Vejlstrup N^5^, Holst LM^1^, Juul K^6^, Smerup MH^7,8^,

^§^Gjedsted J^1,8^, ^§^Ravn, HB^3,4^.

^§^ Shared last authorship.

^1^Department of Cardiothoracic Anesthesiology, Rigshospitalet, Copenhagen University Hospital, Copenhagen, Denmark

^2^Faculty of Health and Medical Sciences, University of Copenhagen, Copenhagen, Denmark

^3^Department of Anesthesiology and Intensive Care, Odense University Hospital, Odense, Denmark

^4^Department of Clinical Research, University of Southern Denmark, Odense, Denmark

^5^Department of Cardiology, Rigshospitalet, Copenhagen University Hospital, Copenhagen, Denmark

^6^Department of Pediatric Cardiology, Rigshospitalet, Copenhagen University Hospital, Copenhagen, Denmark

^7^Department of Cardiothoracic Surgery, Rigshospitalet, Copenhagen University Hospital, Copenhagen, Denmark

^8^Department of Clinical Medicine, University of Copenhagen, Copenhagen, Denmark

For information regarding this article, contact the corresponding author.

Email: [Jonas.Kronborg@regionh.dk](mailto:Jonas.Kronborg@regionh.dk)

| **Stage** | **Serum creatinine criteria** | | **Urine output criteria** *(ml/kg/h)* | |
| --- | --- | --- | --- | --- |
|  | **Pediatric** | **Neonatal modifications** | **Pediatric** | **Neonatal modifications** |
| 1 | ≥0.3 mg/dl rise within 48 h or ≥1.5–1.9 × rise from baseline**^a^** | ≥0.3 mg/dl rise within 48 h or ≥1.5–1.9 × rise from reference**^b^** | ≤ 0.5 for 6-12h | ≤ 1 for 24 h |
| 2 | ≥2.0–2.9 × rise from baseline | | ≤ 0.5 for ≥12 h | ≤ 0.5 for 24 h |
| 3 | ≥3.0 × rise from baseline or ≥4.0 mg/dl or KRT or eGFR <35 ml/min per 1.73 m^2^ | ≥3.0 × rise from reference or ≥2.5^c^ or KRT | ≤ 0.3 for 24 h or  anuria for ≥ 12h | ≤ 0.3 for 24 h |

**Table S1** Kidney Disease: Improving Global Outcomes (KDIGO) Acute Kidney Injury (AKI) Classification including neonatal modifications**.**

**^a^**Baseline within the prior 3 months. A rise from baseline must be known or presumed to have occurred in the prior 7 days. ^b^ Previous lowest SCr in the prior 7 days (a rolling baseline) ^c^ Equivalent to an eGFR <10 ml/min per 1.73 m^2^ in neonates. Abbreviations: KRT, Kidney replacement treatment

|  | | **Neonates**  *n=61* | **Infants**  *n= 167* | **p-value** |
| --- | --- | --- | --- | --- |
| **Age** (days) | | 8.0 (6.0–10.0) | 127.0 (81.0–192.0) | N/A |
| **Weight** (kg) | | 3.5 (3.2–3.9) | 5.6 (4.5–6.8) | N/A |
| **CPB duration** (min) | | 129.0 (108.0–182.0) | 92.0 (69.5–141.0) | N/A |
| **Clamp time** | | 70.5 (49.8–107.5) | 57.0 (42.0–89.0) | N/A |
| **RACHS1 Category (**numerical) | | 4.0 (3.0–4.0) | 2.0 (2.0 – 2.0) | N/A |
| **RACHS-1 Category**  (categorical) | **1-2** | 2 (3.3%) | 126 (75.4%) | N/A |
|  | **3-4** | 55 (90.2%) | 41 (24.6%) |  |
|  | **5-6** | 4 (6.6%) | 0 (0%) |  |
| **Perioperative data** | | | | |
| **Darrow glucose** (ml kg^-1^) | | 66.0 (50.0–82.2) | 56.1 (39.6–76.5) | 0.038 |
| **IV Glucose** (ml kg^-1^) | | 43.5 (16.4–70.5) | 12.2 (4.7–26.9) | <0.001 |
| **Free water intake** (ml kg^-1^) | | 85.7 (67.4–129.9) | 55.6 (38.3–79.4) | <0.001 |
| **Blood products** (ml kg^-1^) | | 73.2 (49.4–117.4) | 31.8 (19.9–51.9) | <0.001 |
| **RBC transfusions** (ml kg^-1^) | | 38.1 (23.6–63.2) | 16.9 (8.4–28.3) | <0.001 |
| **Cryoprecipitate** (ml kg^-1^) | | 17.1 (11.3–26.0) | 6.6 (0.0–12.7) | <0.001 |
| **Pooled thrombocytes** (ml kg^-1^) | | 16.8 (11.5–28.3) | 9.0 (2.9–15.8) | <0.001 |
| **Intraoperative bleeding** (ml kg^-1^ h^-1^) | | 11.5 (5.8–23.2) | 4.6 (2.5–8.8) | <0.001 |
| **Postoperative bleeding** (ml kg^-1^ h^-1^) | | 1.2 (0.9–2.0) | 0.9 (0.7–1.4) | <0.001 |
| **Furosemide dose** (mg kg^-1^) | | 12.5 (4.7–20.3) | 6.0 (1.7–11.3) | <0.001 |
| **Diuresis^§^** (ml kg^-1^ h^-1^) | | 7.0 (5.8–8.8) | 4.3 (3.5–5.3) | <0.001 |
| **Human albumin** (g kg^-1^) | | 8.5 (6.7–10.0) | 4.7 (3.6–5.9) | <0.001 |
| **Cumulative-FB POD0**(%) | | 0.1 (-3.1–5.3) | 1.6 (-0.3–3.8) | 0.128 |
| **Cumulative-FB POD1**(%) | | -3.1 (-7.6–0.6) | 0.3 (-2.1–3.2) | <0.001 |

**Table S2** Overall demographics, patient characteristics, and perioperative data.

Blood products comprise the total volume of red blood cells, cryoprecipitate, and pooled platelets.

Data presented as median (IQR) or n (%). Abbreviations: AKI; Acute kidney injury, Cumulative-FB; Cumulative Fluid Balance**,** N/A; Not Applicable, IV; intravenous, POD; Postoperative day, RACHS-1; Risk Adjustment for Congenital Heart Surgery 1, RBC; Red blood cells.

|  | **No AKI**  *n= 23* | **AKI**  *n= 38* | **p-value** |
| --- | --- | --- | --- |
| **Human albumin**  (g kg^-1^) | 7.1  (6.0 – 9.0) | 8.8  (7.3 – 10.9) | **0.019** |
| **Biarbonate**  (mEq kg^-1^) | 6.0  (4.3 – 8.4) | 8.1  (6.4 – 9.6) | **0.046** |
| **Daily fluid balance POD0**  (ml kg^-1^) | 0.8  (-33.4 – 46.4) | 6.3  (-29.3 – 58.4) | 0.582 |
| **Daily fluid balance POD1**  (ml kg^-1^) | -40.6  (-53.4 – -26.7) | -46.1  (-75.7 – -27.9) | 0.162 |
| **Dysnatremia** | | | |
| **Normonatremia** | 9 (39.1%) | 13 (34.2%) | 0.144 |
| **Hyponatremia** | 5 (21.7%) | 17 (44.7%) |  |
| **Hypernatremia** | 9 (39.1%) | 8 (21.1%) |  |
| **KDIGO** | | | |
| **Stage 0** | 23 (100.0%) | 0 (0.0%) | N/A |
| **Stage 1** | 0 (0.0%) | 26 (68.4%) |  |
| **Stage 2** | 0 (0.0%) | 4 (10.5%) |  |
| **Stage 3** | 0 (0.0%) | 8 (21.1%) |  |

**Table S3** In neonates, accumulated volume/doses intra- and postoperatively (first up to 48 postoperative hours).

Data presented as median (IQR) or n (%). Abbreviations: AKI; Acute kidney injury, KDIGO; Kidney disease improving global outcomes, N/A; Not Applicable, POD; Postoperative day.

|  | **No AKI**  *n= 65* | **AKI**  *n= 102* | **p-value** |
| --- | --- | --- | --- |
| **Human albumin**  (g kg^-1^) | 4.8  (3.8 – 5.6) | 4.7  (3.6 – 6.0) | 0.929 |
| **Biarbonate**  (mEq kg^-1^) | 3.7  (1.0 – 5.6) | 4.1  (2.6 – 5.5) | 0.436 |
| **Daily fluid balance POD0**  (ml kg^-1^) | 11.9  (-14.0 – 33.5) | 16.6  (1.8 – 42.1) | 0.054 |
| **Daily fluid balance POD1**  (ml kg^-1^) | -11.8  (-26.9 – 14.9) | -21.5  (-41.5 – 6.7) | 0.046 |
| **Dysnatremia** | | | |
| **Normonatremia** | 37 (56.9%) | 31 (30.4%) | **<0.001** |
| **Hyponatermia** | 15 (26.2%) | 30 (29.4%) |  |
| **Hypernatremia** | 11 (16.9%) | 41 (40.2%) |  |
| **KDIGO** | | | |
| **Stage 0** | 65(100%) | 0 (0.0%) | N/A |
| **Stage 1** | 0 (0.0%) | 58 (56.9%) |  |
| **Stage 2** | 0 (0.0%) | 39 (38.2%) |  |
| **Stage 3** | 0 (0.0%) | 5 (4.9%) |  |

**Table S4** In infants, accumulated volume/doses intra- and postoperatively (first up to 48 postoperative hours).

Data presented as median (IQR) or n (%). Abbreviations: AKI; Acute kidney injury, KDIGO; Kidney disease improving global outcomes, N/A; Not Applicable, POD; Postoperative day.

**Table S5 Comparison of infants excluded and included in the multivariate analysis.**

Data were missing on three specific variables: intraoperative bleeding (n = 34), cryoprecipitate administration (n = 5), and red blood cell (RBC) transfusion (n = 1). Data are presented as median (IQR) or n (%). Abbreviations: AKI; Acute kidney injury, CPB; Cardiopulmonary bypass, RACHS-1; Risk Adjustment for Congenital Heart Surgery 1, POD; Postoperative day.

|  | **Excluded**  *n= 40* | **Included**  *n= 188* | **p-value** |
| --- | --- | --- | --- |
| **Age** *(days)* | 89.0 (37.8 – 147.5) | 92.0 (19.2 – 181.2) | 0.956 |
| **Neonate** | 10 (25.0%) | 51 (27.1%) | 0.937 |
| **Weight** (kg) | 4.9 (3.6 – 6.0) | 4.8 (3.8 – 6.5) | 0.693 |
| **Sex** (male) | 20 (50.0%) | 114 (60.6%) | 0.287 |
| **CPB time** *(minutes)* | 97.5 (68.8 – 141.0) | 112.5 (79.0 – 153.0) | 0.262 |
| **AKI** | 25 (62.5%) | 115 (61.2%) | 1.0 |
| **Human albumin** (g kg^-1^) | 5.4 (3.8 – 7.3) | 5.4 (4.0 – 7.3) | 0.928 |
| **IV Glucose** (ml kg^-1^) | 1.5 (1.2 – 2.0) | 1.4 (1.1 – 2.1) | 0.793 |
| **Free water intake** (ml kg^-1^) | 68.0 (46.0 – 85.2) | 62.1 (41.0 – 91.9) | 0.857 |
| **Postoperative bleeding** (ml kg^-1^ h^-1^) | 1.0 (0.8 – 1.3) | 1.0 (0.7 – 1.5) | 0.9 |
| **Furosemide dose** (mg kg^-1^) | 6.7 (2.2 – 18.2) | 7.3 (2.2 – 15.1) | 0.742 |
| **Diuresis** (ml kg^-1^ h^-1^) | 4.5 (3.4 – 6.8) | 4.9 (3.9 – 6.4) | 0.367 |
| **Cumulated fluid balance POD0** | 1.6% (-0.2 – 5.3) | 1.2% (-1.0 – 3.8) | 0.283 |
| **Cumulated fluid balance POD1** | 1.5% (-2.9 – 3.8) | -0.8% (-3.1 – 2.1) | 0.125 |
| **RACHS-1 category** | | | |
| **1-2** | 21 (52.5%) | 107 (56.9%) | 0.327 |
| **3-4** | 19 (47.5%) | 77 (41.0%) |  |
| **5-6** | 0 (0.0%) | 4 (2.1%) |  |
| **Dysnatremia** | | | |
| **Normonatremia** | 19 (47.5%) | 71 (37.8%) | 0.507 |
| **Hyponatremia** | 11 (27.5%) | 58 (30.9%) |  |
| **Hypernatremia** | 10 (25.0%) | 59 (31.4%) |  |


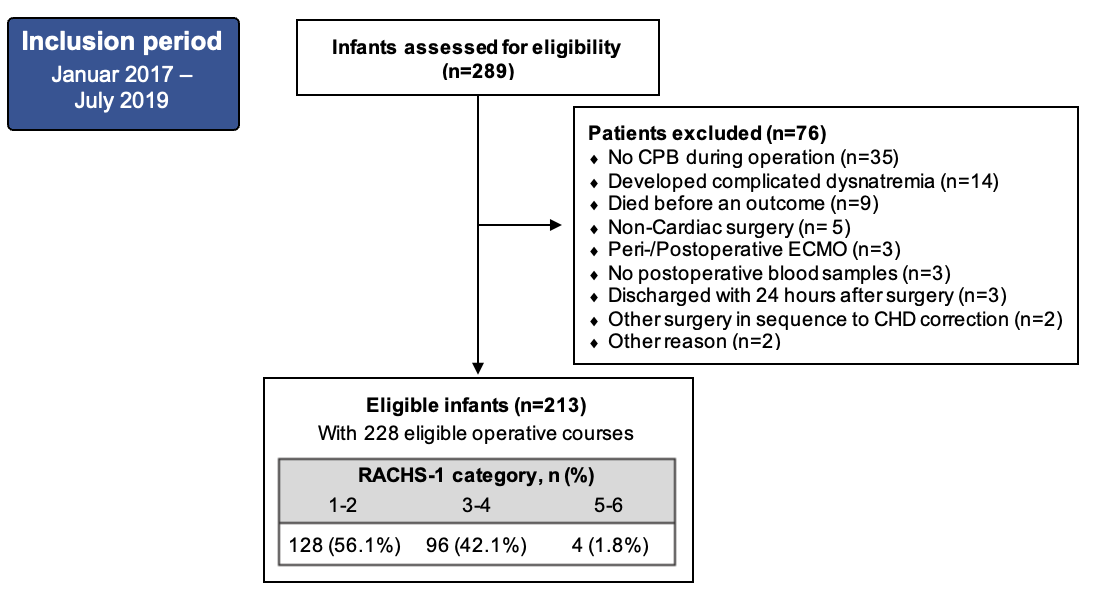


**Fig. S1** STROBE diagram of the included and excluded infants in the study.

Abbreviations: CHD; Congenital heart disease, CPB; Cardiopulmonary bypass, RACHS-1; Risk Adjustment for Congenital Heart Surgery 1.
