# Supplementary material for: Impact of acute kidney injury and dysnatremia on length of stay in infants after cardiac surgery
Source: Pediatr Nephrol. 2025 Jun 14;40(10):3281–91. doi: 10.1007/s00467-025-06846-7 (PMC12402015; doi:10.1007/s00467-025-06846-7)
Supplement: Supplementary file 2 — Graphical abstract (PPTX 432 KB) [file 467_2025_6846_MOESM2_ESM.pptx]

## Slide 1
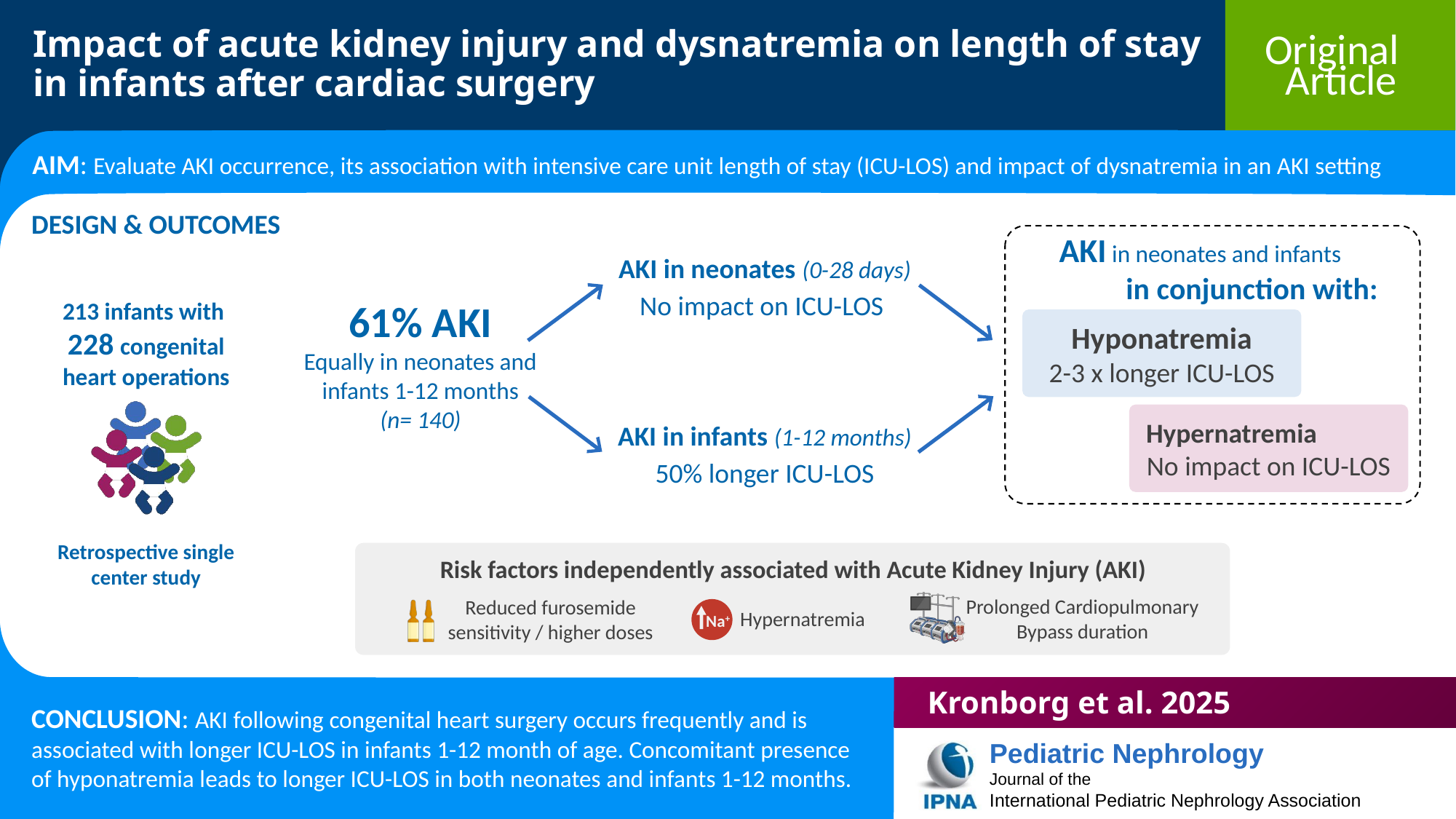

Impact of acute kidney injury and dysnatremia on length of stay
in infants after cardiac surgery
AIM: Evaluate AKI occurrence, its association with intensive care unit length of stay (ICU-LOS) and impact of dysnatremia in an AKI setting
DESIGN & OUTCOMES
AKI in neonates and infants
 in conjunction with:
AKI in neonates (0-28 days)
No impact on ICU-LOS
61% AKI
Equally in neonates and infants 1-12 months
(n= 140)
Hyponatremia
 2-3 x longer ICU-LOS
Hypernatremia No impact on ICU-LOS
AKI in infants (1-12 months)
50% longer ICU-LOS
213 infants with
228 congenital heart operations
Retrospective single center study
Risk factors independently associated with Acute Kidney Injury (AKI)
Prolonged Cardiopulmonary
Bypass duration
Reduced furosemide sensitivity / higher doses
Na+
Hypernatremia
Kronborg et al. 2025
CONCLUSION: AKI following congenital heart surgery occurs frequently and is associated with longer ICU-LOS in infants 1-12 month of age. Concomitant presence of hyponatremia leads to longer ICU-LOS in both neonates and infants 1-12 months.
